# Immortalization and Characterization of Rat Lingual Keratinocytes in a High-Calcium and Feeder-Free Culture System Using ROCK Inhibitor Y-27632

**DOI:** 10.3390/ijms22136782

**Published:** 2021-06-24

**Authors:** Zixing Chen, Wenmeng He, Thomas Chun Ning Leung, Hau Yin Chung

**Affiliations:** 1 School of Life Sciences, The Chinese University of Hong Kong, Hong Kong, China; joshuachen@link.cuhk.edu.hk (Z.C.); chunningtleung@cuhk.edu.hk (T.C.N.L.); 2 Division of Science and Technology, Beijing Normal University-Hong Kong Baptist University United International College, Zhuhai 519087, China; wenmenghe@uic.edu.hk; 3 State Key Laboratory of Agrobiotechnology, The Chinese University of Hong Kong, Hong Kong, China

**Keywords:** lingual keratinocytes, immortalization, ROCK inhibitor, Y-27632, high calcium, feeder-free, taste bud cells, proteomics

## Abstract

Cultured keratinocytes are desirable models for biological and medical studies. However, primary keratinocytes are difficult to maintain, and there has been little research on lingual keratinocyte culture. Here, we investigated the effect of Y-27632, a Rho kinase (ROCK) inhibitor, on the immortalization and characterization of cultured rat lingual keratinocyte (RLKs). Three Y-27632–supplemented media were screened for the cultivation of RLKs isolated from Sprague–Dawley rats. Phalloidin staining and TUNEL assay were applied to visualize cytoskeleton dynamics and cell apoptosis following Y-27632 removal. Label-free proteomics, RT-PCR, calcium imaging, and cytogenetic studies were conducted to characterize the cultured cells. Results showed that RLKs could be conditionally immortalized in a high-calcium medium in the absence of feeder cells, although they did not exhibit normal karyotypes. The removal of Y-27632 from the culture medium led to reversible cytoskeletal reorganization and nuclear enlargement without triggering apoptosis, and a total of 239 differentially expressed proteins were identified by proteomic analysis. Notably, RLKs derived from the non-taste epithelium expressed some molecular markers characteristic of taste bud cells, yet calcium imaging revealed that they rarely responded to tastants. Collectively, we established a high-calcium and feeder-free culture method for the long-term maintenance of RLKs. Our results shed some new light on the immortalization and differentiation of lingual keratinocytes.

## 1. Introduction

Keratinocytes are the principal cells of the epidermis which form a rigid stratified structure through differentiation and provide the body’s outermost barrier against environmental stimuli. Although keratinocyte culture is an important tool for many dermatological and biological studies [1,2,3], long-term maintenance of keratinocytes has been difficult because keratinocytes tend to undergo terminal differentiation and have very short replicative life spans in culture [4,5,6]. Therefore, many attempts have been made to enhance the proliferative capabilities of cultured keratinocytes, which include co-culturing with feeder cells [7,8], using serum-free and low-calcium media [9,10,11], utilizing tissues from neonatal animals for primary culture [4,5], and using conditioned medium [5]. Although these methods managed to extend the propagation of keratinocytes to a certain degree, the complexity and availability of the specialized culture conditions are still substantial hurdles to starting keratinocyte culture.

Lingual keratinocytes refer to the predominant cells located in the epithelial layer of the tongue. In mammals, the surface of the tongue is composed of a multi-layered sheet of keratinocytes. During development, the actively proliferating keratin (Krt) 14^+^/5^+^ basal cells undergo differentiation and gradually migrate upward to become Krt 8^+^ taste bud cells or krt 13^+^ non-taste epithelial cells [12,13,14,15]. Compared to skin and gingival keratinocytes which have been studied extensively [1,4,8,15,16], there was only a smattering of studies trying to cultivate and characterize lingual keratinocytes. These studies all required some of the aforementioned complicated culture methods [11,17,18,19], while the characteristics of the cultured keratinocytes remain poorly studied.

Recently, some studies have demonstrated that Y-27632, a selective inhibitor of Rho-associated protein kinase (ROCK), effectively prolonged the lifespans of some epithelial cells in the presence of feeder cells [20,21,22,23,24]. However, due to the limited numbers of studies on lingual epithelial cells, it is unclear whether ROCK inhibitor is effective in the long-term maintenance of lingual keratinocytes. Therefore, the objectives of this study are (1) to examine the effect of ROCK inhibitor Y-27632 on the long-term culture of rat lingual keratinocytes (RLKs) and (2) to characterize the cultured keratinocytes in terms of gene expression and cellular functions. We found that Y-27632 enabled conditional immortalization of rat lingual keratinocytes in a modified growth medium that was originally designed for the culture of taste bud cells. The keratinocytes maintained high proliferative capacities in a high-calcium condition without the presence of feeder cells. Our study also showed that Y-27632 played an important role in regulating the cytoskeletal dynamics, proliferation, and growth of the keratinocytes. Interestingly, non-taste lingual epithelia could give rise to quasi-taste bud cells that expressed some of the molecular markers characteristic of taste bud cells. To the best of our knowledge, this is the first study to immortalize and characterize cultured lingual keratinocytes in high-calcium and feeder-free culture conditions.

## 2. Results

### 2.1. Isolation of RLKs and Screening of Culture Media

**Figure 1:**
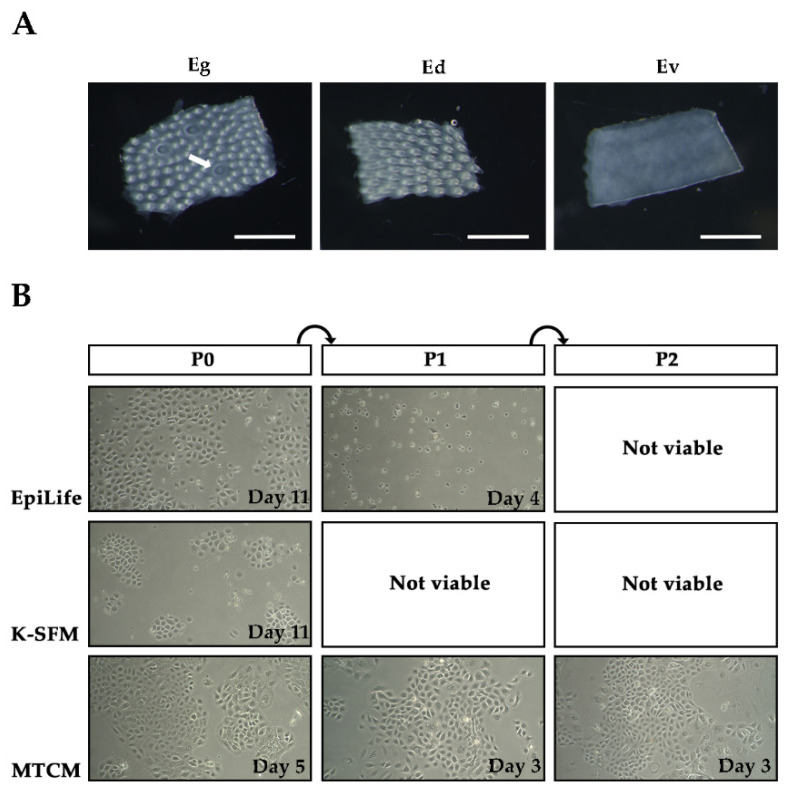
Photographs of rat lingual epithelia and rat lingual keratinocytes (RLKs). (**A**) A dorsal lingual epithelium containing both fungiform papillae and non-taste filiform papillae (Eg); a dorsal lingual epithelium containing only non-taste filiform papillae (Ed); a ventral lingual epithelium without any lingual papillae (Ev). The white arrow indicates a fungiform taste papilla. Scale bars, 500 μm. (**B**) Representative images of lingual keratinocytes cultured in EpiLife, K-SFM, and MTCM growth media. P0 stands for primary culture whereas P1 and P2 represent passage 1 and 2, respectively. Images were captured under 10× magnification. The time points at the bottom-right corners of each panel indicate the time when the photographs were taken after primary culture or subcultures.

After treating with collagenase and dispase mixture for ~20 min at room temperature, the lingual epithelia from the dorsal and ventral side of the tongue could be easily peeled off (Figure 1A). To screen a growth medium suitable for the long-term expansion of RLKs, we tried two different commercially available media—EpiLife and K-SFM—that were widely used for keratinocyte culture and one high-calcium taste cell culture medium (MTCM) modified from previous studies (Table 1). We first tested these media without Y-27632 supplementation. As expected, although some of the RLKs could grow after primary culture, almost all of them died after the first subculture (data not shown). Therefore, the culture media were then supplemented with 10 μM ROCK inhibitor Y-27632 in accordance with previous literature [20,21,22,25]. We found that RLKs could attach and grow in all three types of media during primary culture, and these cells adopted a polygonal appearance typical of epithelial cells (Figure 1B). However, their proliferation rates differed significantly in different growth media even when seeding at the same density (~5000 cells/cm^2^). It took 5 and 11 days for the cells to reach ~70% confluence in MTCM and EpiLife, respectively. By contrast, cells cultured in K-SFM were unable to reach 70% confluence for as long as 30 days. Moreover, although the cells were able to grow in EpiLife and K-SFM during primary culture, most of the cells died after the first subculture and could not be continuously cultured for more than 2 passages (Figure 1B). Only cells cultured in MTCM could be continuously passaged without losing their replicative potential. These results suggested that the ROCK inhibitor alone did not necessarily enable the continuous propagation of keratinocytes, and a proper formulation of the growth medium is also essential for the long-term maintenance of keratinocytes in the absence of feeder cells. Notably, the RLKs proliferated rapidly in MTCM. A piece of the lingual epithelium as small as ~1 mm^2^ was capable of giving rise to a large number of primary cells sufficient for serial cultivation in less than one week. Therefore, MTCM was chosen to culture the RLKs in subsequent experiments.

### 2.2. Conditional and Reversible Immortalization of RLKs by Y-27632

So far, the RLKs derived from both dorsal and ventral lingual epithelia have been continuously passaged more than 80 times (approximately 250 population doublings) in MTCM without visible changes in morphology (Supplementary Figure S1). These cells far exceeded the Hayflick Limit and could therefore be considered immortalized [29].

**Figure 2:**
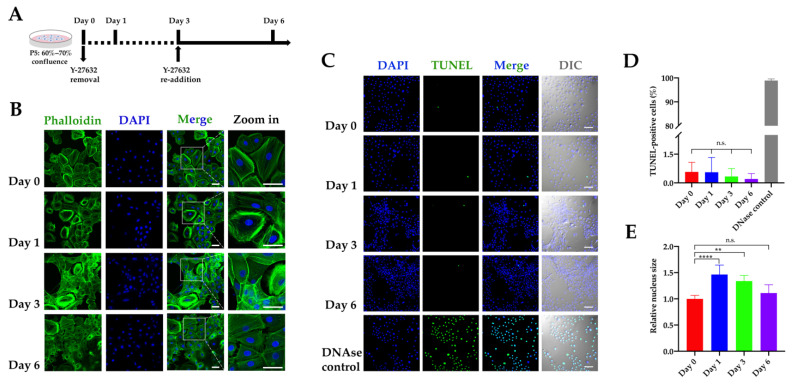
Effect of Y-27632 on the cytoskeletal dynamics and apoptosis of RLKs. (**A**) Experimental scheme. Cells were continuously cultured in MTCM with Y-27632 till passage 5. Then, Y-27632 was removed from the culture medium when cells reached 60–70% confluence on day 0 and re-added to the culture medium on day 3. Cells on day 0, 1, 3, and 6 were harvested for analysis. (**B**) Cytoskeletal reorganization in RLKs following Y-27632 removal and re-addition. Actin filaments were stained with Phalloidin-iFluor 488 (green) and nuclei were stained with DAPI (blue). Scale bars, 50 μm. (**C**) Representative images of TUNEL-positive nuclei (green) and DAPI-stained nuclei (blue) in RLKs at different time points. DNAse-treated cells were used as a positive control. Scale bar, 100 μm. (**D**) Quantification of TUNEL-positive cells (*n* = 6 six random separate fields; n.s. = non-significant; one-way ANOVA). (**E**) Relative nucleus area of RLKs at different time points. Data were normalized with the average nucleus size on Day 0 (*n* = 6 six random separate fields; **** *p* < 0.0001, ** *p* = 0.0017, n.s. = non-significant; one-way ANOVA).

Consistent with previous findings [24,30], we found that the effect of Y-27632 on RLKs was conditional. When Y-27632 was removed from the culture medium, significant morphological changes and cytoskeletal remodeling could be observed in the cells. As shown in Figure 2A–C, RLKs became flattened and gradually formed irregular sheet-like structures after the removal of Y-27632. Meanwhile, the removal of the ROCK inhibitor resulted in a reduced growth rate, although it did not completely abolish cell proliferation (Supplementary Figure S2). Without Y-27632, RLKs could hardly attach to culture disks after subculture and were unmaintainable (data not shown). Notably, when the ROCK inhibitor was re-added to the cell culture, some of the cells reverted to actively proliferative states and gave rise to daughter cells that displayed polygonal shapes again. These results showed that the immortalization of RLKs by Y-27632 was conditional and reversible.

One of the key pathways by which ROCK inhibitor regulates cell survival is through inhibition of cell apoptosis [31,32]. However, although morphological changes were found following the removal of Y-27632, we did not observe significant cell shrinkage or membrane blebbing indicative of cell apoptosis, suggesting that Y-27632 may not regulate the RLKs by inhibiting apoptosis. To confirm this conjecture, a transferase-mediated dUTP-fluorescein nick end labeling (TUNEL) assay was performed to identify and quantify the apoptotic cells. As expected, there was no significant difference in the percentage of apoptotic cells between different experiment groups (Figure 2C,D), suggesting that the main role of ROCK inhibitor in lingual keratinocyte maintenance was not apoptosis inhibition. Interestingly, we also found that RLKs without Y-27632 exhibited enlarged nuclei, a phenotype of cellular senescence [33], which were also reversible upon the re-addition of Y-27632 (Figure 2E).

### 2.3. Proteome Profiling of RLKs in the Presence and Absence of Y-27632

**Figure 3:**
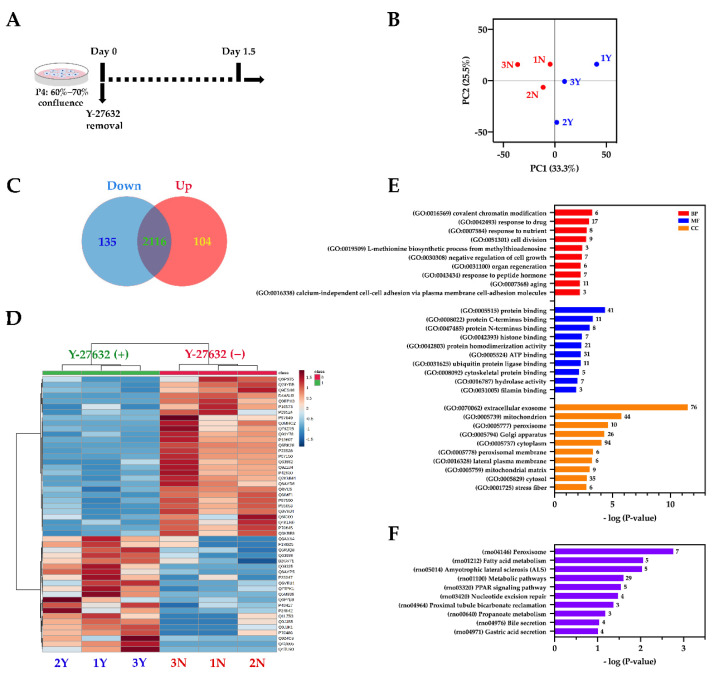
Comparative proteomic analysis of RLKs in the presence and absence of Y-27632. (**A**) Experimental scheme. Cells were continuously cultured in MTCM with Y-27632 till passage 4. Then, Y-27632 was removed from the culture medium when cell culture reached 60–70% confluence on day 0. Cells on day 0 and day 1.5 were harvested for analysis. (**B**) Score plot of principal component analysis (PCA). RLKs treated with (blue dots, 1Y–3Y) or without (red dots, 1N–3N) Y-27632 were separated into two groups (*n* = 3 biological replicates). (**C**) Venn diagram showing the numbers of upregulated (Up) and downregulated (Down) proteins following the removal of Y-27632. (**D**) Hierarchical clustering showing the top 50 differentially expressed proteins. Ward clustering algorithm, Euclidean distance measure, and autoscale features were used. (**E**) Gene Ontology (GO) enrichment analysis of the 239 differentially expressed proteins. The 10 most significantly enriched GO terms in biological process (BP), molecular function (MF), and cell component (CC) are presented. The number on the right side of each column represents the number of proteins associated with the corresponding GO term. (**F**) KEGG pathway enrichment analysis of the 239 differentially expressed proteins. The 10 most significantly enriched pathways are presented. The number on the right side of each column represents the number of proteins associated with the corresponding KEGG pathway.

Although Y-27632 is effective in maintaining keratinocytes, the mechanism by which Y-27632 induces immortalization is still unclear. To further study the effect of Y-27632 on RLKs, we conducted a comparative proteomic analysis to examine the proteome profiles of the RLKs in the presence and absence of Y-27632. The RLKs treated with or without Y-27632 for 36 h (Figure 3A) were subjected to proteomic analysis using the filter-aided sample preparation (FASP) method [34]. LC-MS/MS results showed that a total of 2355 proteins were identified with high confidence (experimental q-value < 1%). As shown in Figure 3B, the principal component analysis (PCA) showed that the RLKs treated with or without Y-27632 could be separated into two groups, with the first two components explaining 58.8% of the data variance, suggesting that the removal of ROCK resulted in significant proteomic changes. To identify the differentially expressed proteins, an adjusted *p*-value < 0.01 and a fold change ≥2 were set as the cut-offs. As shown in Figure 3C, a total of 135 proteins were found downregulated and 104 proteins were found upregulated following the removal of Y-27632. The heatmap in Figure 3D displays the top 50 differentially expressed proteins. To gain further insight into the biological implications of the differentially expressed proteins, Gene Ontology (GO) and Kyoto Encyclopedia of Genes and Genomes (KEGG) pathway enrichment analyses were performed using the DAVID database [35]. Interestingly, numerous GO terms related to cell proliferation or growth such as cell division (GO:0051301), negative regulation of cell growth (GO:0030308), and aging (GO:0007568) were enriched (Figure 3E). KEGG pathways enrichment analysis suggested that the differentially expressed proteins may be associated with peroxisome (rno04146), metabolic pathways (rno01100), and nucleotide excision repair (rno03420) (Figure 3F).

### 2.4. Characterization of RLKs

**Figure 4:**
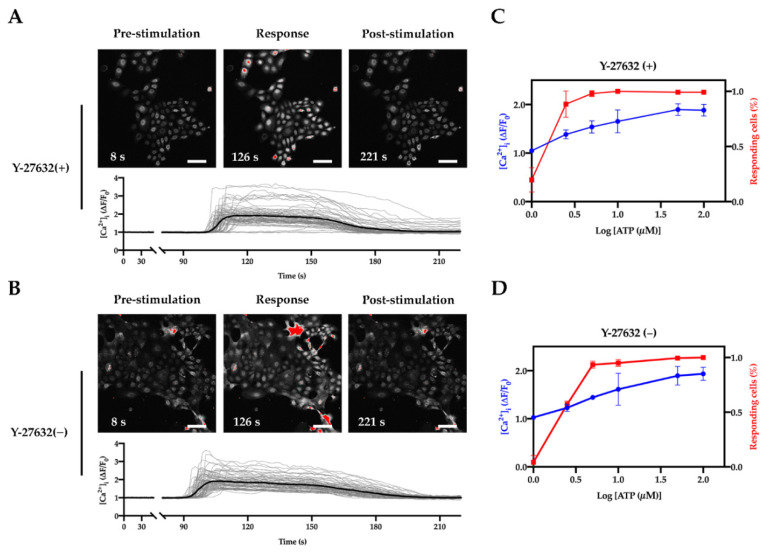
ATP-induced calcium responses in RLKs. (**A**,**B**) Representative images and traces showing the changes of cytosolic Ca^2+^ in responses to 50 µM ATP in RLKs treated with Y-27632 (**A**) or without Y-27632 for 1 day (**B**). Individual cells are stained with Fluo-4 and pseudo-colored in white. Red areas indicate cells that have a very high intracellular calcium level to the extent that oversaturation occurs. The gray traces represent the response profiles of individual cells and the black traces represent the average of the responses. Scale bar, 100 μm. Note: The difference in the start time of responses of the cells in panels (**A**,**B**) was due to exogenous inter-sample variances caused by the experimental setup and handling. (**C**,**D**) Dose-dependent responses of RLKs to ATP at different concentrations (1 µM, 2.5 µM, 5 µM, 10 µM, 50 µM, and 100 µM) in the presence (C; *n* = 3 coverslips; total no. of cells analyzed = 247) and absence (D; *n* = 3 coverslips; total no. of cells analyzed = 226) of Y-27632 for 1 day. The cells analyzed were at passage 5.

As there were very few studies trying to cultivate lingual keratinocytes, cultured lingual keratinocytes have remained poorly characterized. Therefore, we applied reverse transcription polymerase chain reaction (RT-PCR) and live-cell calcium imaging to study the gene expression and cellular functions of the RLKs. In mammalian gustatory systems, adenosine triphosphate (ATP) is an important afferent taste transmitter. The ATP released from Type II taste bud cells can also stimulate Type III taste bud cells to induce cellular responses [36]. We first tested the responses of the cultured keratinocytes to different concentrations of ATP using live-cell calcium imaging. As shown in Figure 4, ATP induced calcium response in a dose-dependent manner in both cells treated with or without Y-27632. While cells treated without Y-27632 were less sensitive to ATP at low concentration (<2.5 µM), almost all of the cells responded to ATP at high concentration (≥50 µM) regardless of Y-27632 (Figure 4C,D).

**Figure 5:**
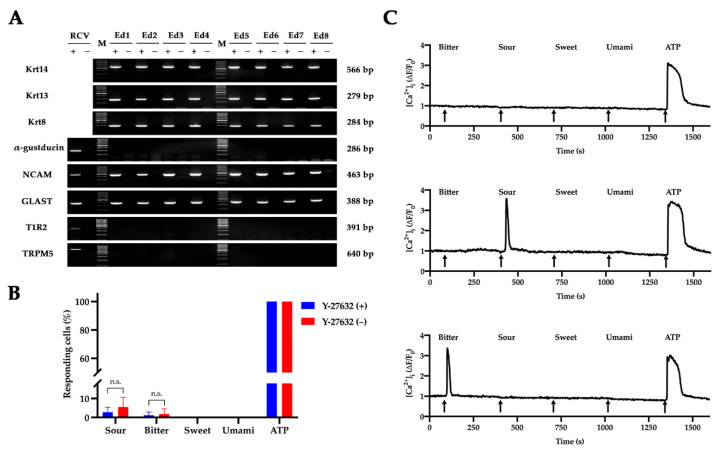
Characterization of cultured keratinocytes derived from non-taste lingual epithelia. (**A**) RT-PCR results showing the presence of krt 14 (basal progenitor marker), krt 13 (post-mitotic non-taste cell marker), krt 8 (pan-taste cell marker), NCAM (type III taste cell marker), and GLAST (type I taste cell marker), and absence of α-gustducin (type II taste cell marker), TRPM5 (type II taste cell marker), and T1R2 (sweet taste receptor) mRNAs in the cultured RLKs. “Ed1” to “Ed8” refer to 8 independent biological replicates of cell culture derived from non-taste dorsal lingual epithelia. “+” and “−” refer to PCR reactions with and without reverse transcriptase. RCV, rat circumvallate papillae tissue. M, 100 bp DNA ladders. The cells used in this experiment were continuously cultured in MTCM with Y-27632 and were harvested for PCR analysis at passage 3. (**B**) Percentage of cells responsive to taste stimuli or ATP. Cells treated with or without Y-27632 for 24–30 h were subjected to calcium imaging analyses. Only cells that responded to ATP during the last stimulation were counted (*n* = 4 coverslips for each treatment; n.s. = non-significant; unpaired *t*-test). The total numbers of cells analyzed were 493 and 373 in the presence and absence of Y-27632, respectively. The stimuli used were: sour (10 mM citric acid), bitter (2 mM denatonium benzoate and 10 µM cycloheximide), sweet (10 mM sucralose and 10 mM acesulfame K), umami (100 mM MSG and 1 mM IMP) and 50 µM ATP. The cells analyzed were at passage 5. (**C**) Representative traces showing the cytosolic Ca^2+^ changes of three cells that responded to ATP only (**top**), sour stimulus and ATP (**middle**), and bitter stimulus and ATP (**bottom**), respectively.

Surprisingly, RT-PCR results showed that the cultured keratinocytes derived from non-taste lingual epithelia (Figure 1A, Ed) expressed some of the taste-related molecules (Figure 5A). Apart from the expression of krt 14, krt 13, and krt 8 which are distinctive markers for progenitors, post-mitotic cells and taste bud cells in the lingual epithelium [15], the keratinocytes in our culture system also expressed the type I taste cell marker GLAST and type III taste cell marker NCAM. However, α-gustducin (a type II taste cell marker), TRPM5 (a type II taste cell marker), and T1R2 (sweet taste receptor) transcripts could not be detected in the cultured cells. To further examine whether the cultured keratinocytes are responsive to taste stimuli, we applied calcium imaging to monitor the changes of intracellular Ca^2+^ in the RLKs upon stimulation of tastants [37]. The calcium imaging results showed that only a very small proportion of cells could respond to bitter and sour stimuli, and none of the cells in our culture system was found to be responsive to sweet and umami stimuli (Figure 5B,C).

**Figure 6:**
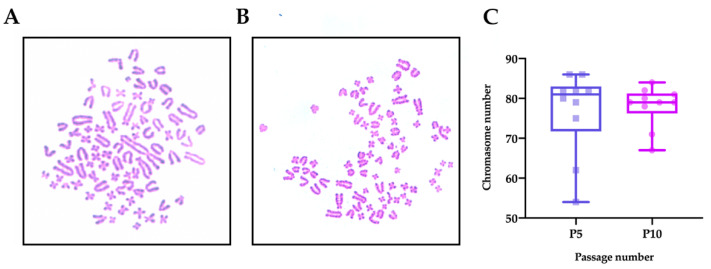
Chromosome analysis of RLKs. (**A**,**B**) Representative images of Giemsa-stained metaphase spreads of RLKs at passage 5 and passage 10, respectively. (**C**) Box-and-whisker plots showing the distribution of the chromosome numbers in RLKs at passage 5 and 10 (*n* = 10 metaphase spread counts). Note: Due to the high number of chromosomes in the cells, the errors in the counting of chromosome numbers were within ± 2 chromosomes.

Finally, we also conducted a cytogenetic analysis to evaluate the genetic stability of the cultured RLKs. In contrast to earlier findings [21,38,39], we found that the RLKs in our culture system were highly aneuploidy. As shown in Figure 6, the modal chromosome number of the RLKs was ~80 (near-tetraploidy), with a few cells having much higher or lower ploidies.

## 3. Discussion

In this research, we found that Y-27632 was a potent drug for the conditional immortalization of rat lingual keratinocytes. This study provides a rapid and simple method for obtaining a large number of rat lingual keratinocytes in vitro. Notably, the culture system adopted in the present study did not require the use of feeder cells. Most previous studies suggested that co-culturing with feeder cells was indispensable for the immortalization of keratinocytes by Y-27632 [20,21,22,24,30]. Only very few studies have managed to establish long-term keratinocyte cultures under specialized culture conditions in the absence of feeder cells [25,39,40,41]. The present study provides another instance demonstrating the possibility of long-term maintenance of keratinocyte without feeder cells. Our results also show that medium composition is an important factor affecting the maintenance of keratinocytes since RLKs were unable to continuously proliferate in the EpiLife and K-SFM media in the presence of Y-27632. Notably, although previous literature suggested that low-calcium (<0.2 mM) medium was important for the long-term cultivation of keratinocytes [10], our results demonstrated that when culturing with the ROCK inhibitor, the lingual keratinocytes were able to continuously proliferate in a high-calcium (~1.4 mM) environment, and therefore the immortalization or long-term cultivation of keratinocytes seems to be independent of the extracellular calcium level.

ROCK is a downstream effector of the Rho GTPases that control a variety of cellular processes including cytoskeletal organization, cell polarity, cell adhesion, membrane transport, and motility [42]. Y-27632, a pharmacological inhibitor of the Rho kinase, was originally identified as a potent inhibitor of apoptosis or anoikis, capable of increasing the survival and cloning efficiency of stem cells [43,44]. However, the present study showed that the removal of the ROCK inhibitor did not significantly affect the percentage of apoptotic cells (Figure 2C). Rather, cells without Y-27632 tended to display phenotypes associated with cellular senescence including flattened appearance and enlarged nuclei (Figure 2B,C,E). These results indicate that Y-27632 may play an important role in fine-tuning the balance between the conditional immortalization and the cellular senescence of keratinocytes. However, the exact mechanism by which Y-27632 induces the immortalization of keratinocytes remains elusive so far. Recently, one study suggested that Y-27632 maintained the proliferative capacity and stemness of human hair follicle stem cells through regulating the ERK/MAPK signaling pathway [45]. By contrast, another study also reported that the inhibition of PAK1-ROCK-Myosin II and TGF-β signaling pathways were crucial for the long-term proliferation of epithelial stem cells in a low-calcium medium [46]. Additionally, the inhibition of the TGF-β pathway has also been demonstrated to be important for the long-term expansion of epithelial basal cells [40]. As the effects of the ROCK inhibitor on the cells are manifold, we are still unable to disentangle the exact immortalization pathway from other cellular pathways that could possibly be affected by Y-27632. This is because the use of 10 µM Y-27632 in the present study may concurrently affect a variety of other cellular processes like cell cycle, cell adhesion, and differentiation through ROCK inhibition [23,42,47,48]. Therefore, for future studies, in order to better understand the mechanism of the Y-27632-induced immortalization, care should be taken to minimize these confounding effects. A dose–response study, for example, may aid in determining the minimum concentration of Y-27632 required for cell immortalization and thereby reduce the impact of other confounding factors. Overall, further investigations with proper controls are needed to elucidate the mechanism by which Y-27632 induces conditional immortalization in keratinocytes.

Label-free quantitative proteomics is a cost-effective and high-throughput method that allows identifying and quantifying relative changes of thousands of proteins in biological samples across multiple experimental conditions [49]. To the best of our knowledge, no previous study has tried to investigate the effect of ROCK inhibitor on keratinocytes at the proteome level. Therefore, we applied the FASP method, a universal and efficient sample preparation technique, to monitor the proteomic changes of the whole-cell lysates of the RLKs. Our results showed that Y-27632 could significantly affect the proteome of the RLKs. The enrichment analyses suggested that the differentially expressed proteins regulated by ROCK inhibitor were mainly associated with cell growth, division and metabolism. The details of the differentially expressed genes enriched in the GO terms and KEGG pathways are listed in Supplementary Table S1. Note that further experiments (e.g., Western blotting) are needed to confirm the changes in the expression of the individual genes. Besides that, from our mass spectrometric data, we failed to identify some low-abundance proteins that may be involved in taste signaling transductions. For example, we could not identify the genes shown in Figure 5A from our proteomic data, indicating that their abundances are below the detection limit of the current method. Future studies that try to purify and concentrate certain cellular components (e.g., membrane proteomics) would help to identify and elucidate the subtle changes of the low-abundance proteins. In addition, isotopic labeling and isobaric tagging techniques can also be utilized to allow more accurate quantification of the proteomic changes [50,51].

In recent years, techniques for culturing taste bud cells and taste organoids have been reported [28,37]. However, as there were very few studies on cultured lingual epithelial cells, the relationship between cultured taste cells and non-taste lingual keratinocytes remains unclarified. In rodent and human gustatory systems, the expression of taste-related molecules on the tongue is restricted to cells within taste buds [15,52]. However, by conducting RT-PCR, we found that non-taste lingual epithelial tissues could give rise to cells expressing markers specific to taste bud cells. We did not ascribe these results to artifacts derived from contamination of taste bud cells during primary culture for the following two reasons: (1) when conducting primary culture, special care was taken to ensure that the non-taste epithelial tissues did not contain any visible taste buds (Figure 1A, Ed), and all the isolated non-taste epithelial tissues were washed twice in fresh Tyrode’s solution before digestion to avoid contamination of potentially dissociated taste bud cells, and (2) more than eight independent biological replicates were performed, and the results were quite consistent (Figure 5A).

Given that both taste bud cells and non-taste lingual epithelial cells are derived from Krt 5^+^/Krt 14^+^ progenitor cells in the basal layer of the lingual epithelium [15,53,54,55,56], it is possible that although the dissected lingual epithelia did not contain visible taste buds, the progenitors in the basal layers retained a certain degree of multipotency and therefore could differentiate into cells that mimicked taste bud cells under the culture condition. Indeed, a previous study found that taste buds consisting of all three differentiated taste cell types could be ectopically induced from non-taste progenitors in vivo, indicating the competency and plasticity of the adult lingual epithelium [57]. Alternatively, the karyotypic instability of the keratinocytes may also have some effect on the expression of the taste-related molecules. Notably, we found that the percentage of cells that could respond to taste stimuli was significantly lower than that of the previously reported cultured taste cells [28,37], and not all the taste-related molecules were found in the cultured keratinocytes (Figure 5A). These results suggest that although some of the taste signaling molecules were expressed in the cultured RLKs, these cells may not express intact taste signaling pathways. Therefore, we call these cells quasi-taste bud cells in the sense that they expressed some, but not all, of the taste cell markers, and they rarely responded to taste stimuli. Future studies that systematically compare these keratinocytes with genuine cultured taste bud cells would help understand the differentiation and development of taste bud cells. Importantly, pharmacological studies utilizing signal transduction inhibitors could be used to further confirm the taste signaling pathways in the cultured keratinocytes and taste bud cells. Transgenic animals and cell sorting techniques could also be applied to help purify the non-taste lingual epithelial cells free of contamination from taste bud cells [37]. Overall, the cell lines established in the present study would serve as useful experiment controls for future study of taste bud cells.

Karyotypic changes are characteristic of many immortal cell lines [58,59]. Although previous studies reported that some keratinocytes retained normal karyotypes in ROCK inhibitor-supplemented growth media [21,38,39], we found that the chromosomal numbers of the RLKs in our culture system were indeed highly variable. One possibility is that the feeder-free culture condition was adopted in the present study, and the absence of feeder cells led to the absence of certain secreted molecules that may contribute to genomic stability. In addition, it is worth noting that although no significant changes in chromosomal numbers were found in the previous literature, further experiments are needed to confirm whether there was any chromosome structure instability (e.g., rearrangement of parts of chromosomes) that could not be easily identified using the traditional Giemsa staining technique [59]. Taken together, it can be inferred that the immortalization of keratinocytes solely by ROCK inhibitor does not guarantee karyotypic stability. Future studies are required to examine the relevance or role of feeder cells in maintaining genomic stability in Y-27632–immortalized keratinocytes.

## 4. Materials and Methods

### 4.1. Materials

Iscove’s Modified Dulbecco’s Medium (IMDM), EpiLife™ Medium, Defined Keratinocytes SFM (K-SFM), mouse EGF recombinant protein (EGF), type IV collagenase, dispase, trypsin-EDTA, antibiotic-antimycotic and gentamicin were obtained from Life Technologies/Gibco (Grand Island, NY, USA). Pierce BCA Protein Assay Kit, TRIzol reagents, M-MLV reverse transcriptase, Fluo-4 AM, Pluronic F-127 and Pierce Peptide Desalting Spin Columns were all purchased from Thermo Fisher Scientific (Waltham, MA, USA). Fetal bovine serum (FBS) was bought from Hyclone (Pasching, Austria). Y-27632 was obtained from ATCC (Manassas, VA, USA). Taq DNA polymerase was purchased from TaKaRa (Tokyo, Japan). FASP Protein Digestion Kit (ab270519) and Phalloidin-iFluor 488 Reagent (ab176753) were purchased from abcam (Cambridge, UK). In Situ Cell Death Detection Kit (11684795910) was obtained from Roche Diagnostics (Indianapolis, IN, USA). DNAse I was from Amersham Biosciences (Piscataway, NJ, USA). All other chemicals were obtained from Sigma-Aldrich (St. Louis, MO, USA) unless otherwise specified.

### 4.2. Primary Culture and Maintenance of RLKs

Sprague–Dawley rats (6 weeks, male and female) were sacrificed by CO_2_ asphyxiation followed by cervical dislocation. Tongues were dissected and immediately placed into ice-cold Ca^2+^-free Tyrode’s solution (140 mM NaCl, 5 mM KCl, 2 mM EGTA, 1 mM MgCl_2_, 10 mM HEPES, 10 mM glucose, 1 mM sodium pyruvate). Then, an enzyme cocktail containing 1.5 mg/mL type IV collagenase and 3 mg/mL dispase was injected between the muscle layer and the lingual epithelium. Following incubation at room temperature for 20 min, the lingual epithelium was gently peeled off and immersed in the Ca^2+^-free Tyrode’s solution. Then, the epithelium was digested by 0.25% trypsin-EDTA at 37 °C for 10 min followed by neutralization with FBS. The cell suspension was then centrifuged, and the cell pellet was resuspended in EpiLife, K-SFM or MTCM (Table 1) and incubated at 37 °C in a humidified atmosphere containing 5% CO_2_. The culture medium was changed every 2–3 days. Cells were passaged when reaching ~70% confluence. In some experiments, special efforts were made to dissect non-taste lingual epithelia devoid of any visible taste papillae (Figure 1A). The non-taste epithelia were rinsed twice with clean Tyrode’s solution before being dissociated and cultured with MTCM. Unless otherwise indicated, all experiments were performed with cells within 5 passages.

### 4.3. Phalloidin Staining

Phalloidin-iFluor 488 Reagent was used to observe structural changes in the cytoskeleton of lingual keratinocytes following the manufacturer’s instructions. Briefly, cells grown on coverslips were fixed with 4% paraformaldehyde in PBS for 20 min at room temperature. The fixed cells were permeabilized with 0.1% Triton X-100 in PBS for 3 min. Then the cells were incubated with phalloidin conjugate working solution for 40 min at room temperature. DAPI at 1 µg/mL was used to stain the nuclei. Fluorescence images were obtained using a Leica TCS SP8 confocal microscope (Wetzlar, Germany).

### 4.4. TUNEL Assay

TUNEL assay was conducted using the In Situ Cell Death Detection Kit following the manufacturer’s instructions. Briefly, cells were fixed with 4% paraformaldehyde in PBS for 60 min and permeabilized with 0.1% Triton X-100 in 0.1% sodium citrate on ice for 2 min. Then, the cells were incubated with TUNEL reaction mixture for 60 min at 37 °C in a humidified chamber in the dark. After washing twice with PBS, cells were counterstained with 1 µg/mL DAPI for 10 min and then analyzed by an Olympus FV1000 IX81-SIM confocal microscope (Melville, NY, USA). A positive control was run in parallel by pretreating permeabilized cells with 1000 U/mL DNase I for 15 min at room temperature prior to labelling procedures. As a negative control, terminal transferase was omitted from the reaction mixture.

### 4.5. Sample Preparation for Mass Spectrometry

Samples were prepared from whole-cell lysates using a modified filter-aided sample preparation (FASP) method as previously described [34]. In brief, cells were lysed in ST buffer (4% SDS, 150 mM Tris-HCl, pH 7.6) and incubated at 95 °C for 5 min. After measuring the protein content using a BCA Protein Assay Kit, DTT was added to the cell lysate to achieve a final concentration of 0.1 M and the mixture was incubated at 60 °C for 1 h. Then, proteins were digested using a FASP Protein Digestion Kit following the manufacturer’s instructions. The digested proteins were desalted using Pierce Peptide Desalting Spin Columns, vacuum concentrated to dryness and stored at −80 °C until mass spectrometric analysis.

### 4.6. Nano-UPLC-MS/MS and Comparative Proteomic Analysis

LC-MS/MS analysis was carried out using a Dionex UltiMate 3000 RSLC system coupled to an Orbitrap Fusion Lumos Tribrid mass spectrometer (Thermo Fisher Scientific; Waltham, MA, USA). Prior to analysis, samples were reconstituted in 0.1% formic acid in water. Then, aliquots containing 1 µg peptides were loaded on an Acclaim PepMap100 C18 trap column (300 μm × 5 mm, 5 µm, 100 Å) and separated on an Acclaim PepMap100 C18 nano column (75 µm × 25 cm, 2 µm, 100 Å). Mobile phase A consisted of 0.1% formic acid and 2% acetonitrile and mobile phase B consisted of 0.1% formic acid and 98% acetonitrile. The gradient elution program was as follows: 0–5 min, A 100%; 5–8 min, A 100% to 94%; 8–48 min, A 94% to 82%; 48–58 min, A 82% to 70%; 58–60 min, A 70% to 20%; 60–65 min, A 20%; 65–66 min, A 20% to 100%; 66–75 min, A 100%. The flow rate was set at 300 nL/min.

The mass spectrometer was operated in Nanospray ionization (NSI) mode with static spray voltage set at 2.0 kV and source temperature at 300 °C. MS scans were acquired in the Orbitrap with a mass resolution of 60,000. The MS scan range was from 375 to 1500 *m*/*z*. A standard AGC target with a maximum injection time of 50 ms was used. Precursor ions with charge state 2–7 and intensity above 50,000 were sampled for fragmentation with 30% collision energy using higher-energy collisional dissociation (HCD) with quadrupole isolation. Precursor isolation windows were set to 1.6 *m*/*z*. Data-dependent MS/MS scans were acquired with a mass resolution of 15,000, respectively. A standard AGC target with a maximum injection time of 250 ms was used. Dynamic exclusion was implemented with a repeat count of 1, and an exclusion duration of 40 s.

All tandem mass (MS/MS) spectra were analyzed by Proteome Discoverer (PD 2.4; Thermo Fisher Scientific; Waltham, MA, USA) with SEQUEST as a search engine. Data were searched against a Swiss-Prot *Rattus norvegicus* (TaxID = 10,116) database. The common Repository of Adventitious Proteins database (cRAP; http://www.thegpm.org/crap/; Date accessed: 12 May 2021) was included to eliminate potential contaminants. Enzyme specificity was set to trypsin, with a maximum of two missed cleavages allowed. The minimum peptide length was set to 6 amino acids. Oxidation of methionine and N-terminal protein acetylation were set as dynamic modifications whereas carbamidomethylation on cysteines was set as a static modification. Precursor ion mass tolerance and fragment ion mass tolerance were set at 10 ppm and 0.02 Da, respectively. Target false discovery rate (FDR) for peptide-spectrum matches (PSMs) and peptides were set at the default values: 0.01 (strict) and 0.05 (relaxed). The label-free quantitation was performed through MS signal intensity with total peptide normalization. The abundances of the proteins, exported by PD 2.4 software, were used for PCA and hierarchical cluster analysis using MetaboAnalyst 5.0 (www.metaboanalyst.org; Date accessed: 12 May 2021) [60]. Proteins with an adjusted *p*-value < 0.01 and a fold change ≥2 were considered differentially expressed. The differentially expressed proteins were subjected to Gene Ontology (GO) and Kyoto Encyclopedia of Genes and Genomes (KEGG) pathway enrichment analyses using the DAVID database (version 6.8) [35].

### 4.7. Reverse Transcription Polymerase Chain Reaction (RT-PCR)

Total RNA was extracted with Trizol reagent and cDNA was reverse transcribed into cDNA using M-MLV reverse transcriptase following the manufacturers’ instructions. PCR was conducted on a Bio-Rad C1000™ thermal cycler (Hercules, CA, USA). The primer sets used were shown in Supplementary Table S2. All the primers were designed to span exon–exon junctions to avoid interference by genomic DNA. Minus reverse transcriptase controls were run in parallel to ensure there were no contaminating genomic DNA. The amplification condition consisted of an initial denaturation at 95 °C for 5 min, 40 cycles of 20 s at 94 °C, 30 s at 58 °C and 80 s at 68 °C, followed by a final extension at 75 °C for 5 min. PCR products were separated on 2% agarose gels, stained with ethidium bromide and visualized using a Bio-Rad ChemiDocTM XRS System (Hercules, CA, USA). The amplified PCR products were confirmed by molecular weights and sequencing (Tech Dragon Limited, Hong Kong, China).

### 4.8. Live-Cell Calcium Imaging

Calcium imaging was applied to measure the responses of cells to a certain stimulus based on the change of intracellular calcium level [37]. Briefly, cells were seeded onto coverslips prior to the experiment. When reaching 60–70% confluence, cells were loaded with 5 µM Fluo-4 AM and 0.02% Pluronic F-127 dissolved in Tyrode’s solution (140 mM NaCl, 5 mM KCl, 2 mM CaCl_2_, 1 mM MgCl_2_, 10 mM HEPES, 10 mM glucose and 1 mM sodium pyruvate) for 30 min. Subsequently, the coverslips were mounted onto an imaging chamber and continuously perfused with Tyrode’s solution. The Tyrode’s solution was delivered to the cells through a tube controlled by a dual-channel peristaltic pump at a flow rate of approximately 2 mL/min. Cells were visualized under an Olympus FV1000 IX81-SIM confocal microscope using the FV10-ASW 4.2 software (Melville, NY, USA). The excitation and emission wavelengths were set at approximately 488 nm and 520 nm, respectively. After starting the recording, cells were first maintained in the Tyrode’s solution for approximately 30–40 s prior to the application of stimuli to make sure that there was no observable baseline shift due to misposition of the imaging chamber. The application of the stimuli was manually done by transferring the pump inlet tube from Tyrode’s solution to the stimuli. The stimuli were prepared by dissolving the following compounds in Tyrode’s solution: ATP (1 µM, 2.5 µM, 5 µM, 10 µM, 50 µM, or 100 µM), bitter (2 mM denatonium benzoate and 10 µM cycloheximide), sour (10 mM citric acid), umami (100 mM MSG and 1 mM IMP), sweet (10 mM sucralose and 10 mM acesulfame K). Stimuli were applied for 1 min, followed by a 4-min rinse period of Tyrode’s solution. Changes in cytosolic Ca^2+^ were presented as relative fluorescence change: ΔF/F_0_ = (F − F_0_)/F_0_, where F_0_ is the average baseline fluorescence signal over a 30 s period at the start of the experiment.

### 4.9. Cytogenetic Analysis

Karyotyping was performed as previously described [61]. Briefly, cells were arrested by 0.1 µg/mL Karyomax colcemid for 2 h at 37 °C. Then, cells were trypsinized and incubated in a 75 mM KCl hypotonic buffer followed by fixation in 3:1 methanol–acetic acid solution. Chromosomes were stained with KaryoMAX Giemsa stain solution and analysed using a Carl Zeiss PALM inverted microscope (Oberkochen, Germany).

### 4.10. Statistical Analysis

Statistical analyses were performed using GraphPad Prism 8 (San Diego, CA, USA) software. All data were expressed as mean ± standard deviation (SD). The statistical difference was set at *p* < 0.05.

## 5. Conclusions

The present study demonstrated that rat lingual keratinocytes could be conditionally immortalized by the ROCK inhibitor Y-27632. To the best of our knowledge, this is the first study to immortalize and characterize lingual keratinocytes in a high-calcium and feeder-free culture system. The established cell line in the present study may facilitate our understanding of regeneration and differentiation of taste bud cells.

## Figures and Tables

**Table 1:** Growth media used for primary culture of rat lingual keratinocytes (RLKs).

	EpiLife	K-SFM	MTCM
**Basal media**	EpiLife™ medium	Defined keratinocytes SFM	IMDM + 10% FBS + 20% MCDB 153 medium
**Supplements**	EpiLife™ defined growth supplement, (10 μM Y-27632) ^1^	Defined keratinocyte SFM growth supplement, (10 μM Y-27632) ^1^	10 ng/mL EGF, (10 μM Y-27632) ^1^
**Antibiotics**	1% antibiotic-antimycotic, 0.015% gentamicin		
**Calcium level**	60 µM	<0.1 mM	~1.4 mM
**References**	[26]	[27]	[1,28]

^1^ In some experiments, Y-27632 was not added to the culture medium. Please refer to the text for details.

## Data Availability

The raw mass spectrometry data and result files have been deposited to the ProteomeXchange Consortium via the JPOST partner repository (https://jpostdb.org/) on 12 May 2021. The data are available at https://repository.jpostdb.org/ accessed on 12 May 2021 with the dataset identifier PXD025812.

## Supplementary Materials

The following are available online at https://www.mdpi.com/article/10.3390/ijms22136782/s1, Figure S1: Representative morphology of cultured RLKs derived from dorsal (A) and ventral (B) lingual epithelia, Figure S2: Effect of Y-27632 on cell proliferation, Table S1: List of differentially expressed genes obtained from enrichment analyses, Table S2: Primer sets used in RT-PCR.
