# Supplementary material for: Immortalization and Characterization of Rat Lingual Keratinocytes in a High-Calcium and Feeder-Free Culture System Using ROCK Inhibitor Y-27632
Source: Int J Mol Sci. 2021 Jun 24;22(13):6782. doi: 10.3390/ijms22136782 (PMC8268148; doi:10.3390/ijms22136782)
Supplement: Supplementary file 1 [file ijms-22-06782-s001.zip › Figure S1-2 & Table S2.pdf]

## Supplementary Information

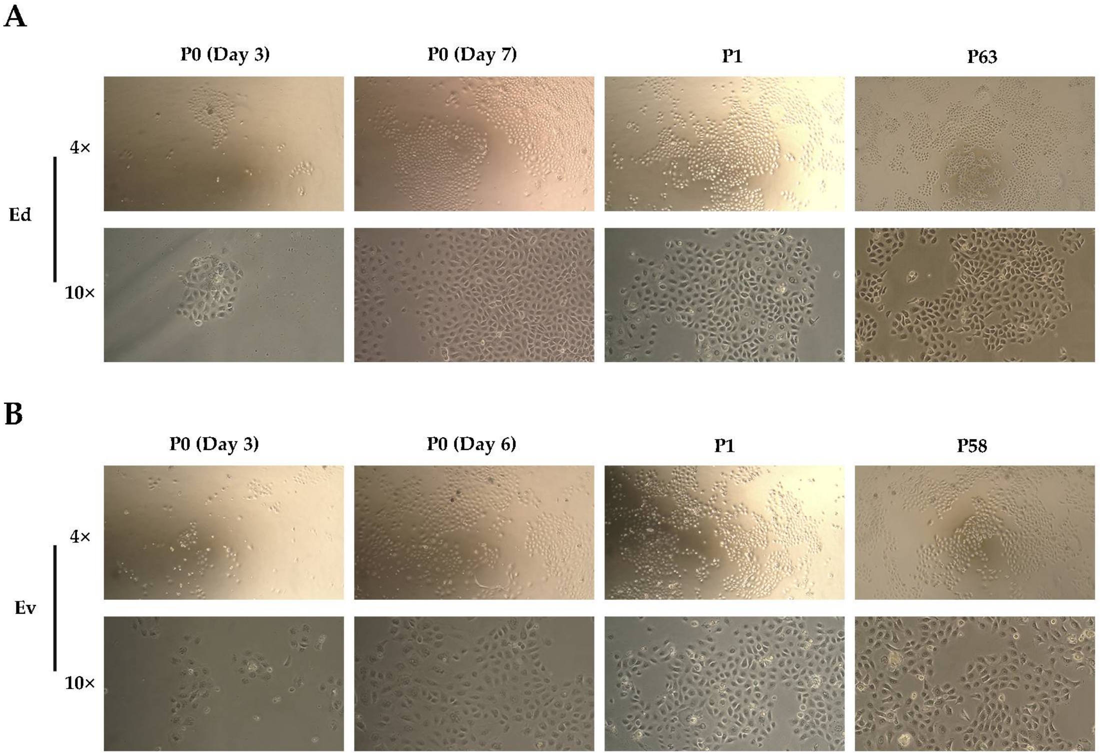


**Figure S1.** Representative morphology of cultured RLKs derived from dorsal (**A**) and ventral (**B**) lingual epithelia. P0 stands for primary culture whereas Pn (n>0) stands for cells at passage n. Images were captured under 10× magnification.

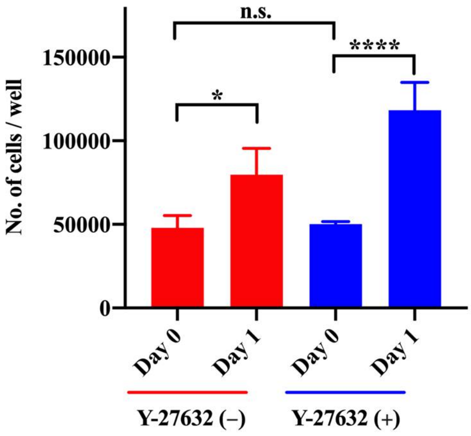


**Figure S2.** Effect of Y-27632 on cell proliferation. Cells were initially seeded onto 48-well culture plates with Y-27632 supplemented. When reaching 60-70% confluence (Day 0), growth media were changed and cells were cultured with or without Y-27632 for another 24 hours (Day 1). The number of cells on day 0 and day 1 were counted with a haemocytometer (n = 4; ****P < 0.0001, *P = 0.0134, n.s. = non-significant; one-way ANOVA).

**Table S2.** Primer sets used in RT-PCR

| Target Gene | Accession No. | Sequence (5'→3') | Size (bp) |
|---|---|---|---|
| Krt 14 | NM_001008751.1 | F: TTCACCTCCTCCAGCTCCAT | 566 |
| | | R: TGCAGGAGGACATTGGCATT | |
| Krt 13 | NM_001004021.1 | F: CATGGTTCCAGGCCAAGAGTG | 279 |
| | | R: CCTGGTTCTGGCACTCCATC | |
| Krt 8 | NM_199370.1 | F: GGAAGCACGGGGATGATCTAC | 284 |
| | | R: GATCTCGATGTCAAGCGCCA | |
| GLAST | NM_019225.2 | F: CGCTCGCTGAGCTGTTACT | 388 |
| | | R: CAAATCCAAGGATTGTACCCACA | |
| $\alpha$-gustducin | NM_173139.2 | F: GCAACCACCTCCATTGTTCT | 286 |
| | | R: AGAAGAGCCCACAGTCTTTGAG | |
| NCAM | NM_031521.1 | F: GTGCAGCCACTGAGTTCAAG | 463 |
| | | R: ATGAGCAGGCCACACTTGTT | |
| TRPM5 | NM_001191896.1 | F: CAAATGTGACATGGTGGCCATC | 640 |
| | | R: GCTCAGGTGGCTGAGCAGGAT | |
| T1R2 | NM_001271266.1 | F: CTACCCGTGGCAGCTACTC | 391 |
| | | R: GTAGGACCACATGGAACCCG | |
